# Mr. Beecher on Hay Fever

**Published:** 1877-11

**Authors:** Henry Ward Beecher


### Mr. BEECHER ON HAY FEVER.

My dear Friend: That you are a novice in the matter of Hay Fever, is very plain from the question that you put. For if you had ever had it, you would never ask, and if you have it, you will never again ask what are the symptoms. Imagine a man asking what are the symptoms by which he might know whether his nose had been pulled, or whether he had been kicked down stairs, or whether some one had thrown over him the unsavory contents of a bucket.

The Hay Fever is all symptoms.

You notice some evening that your eyes are suddenly weak. The light disturbs them. All in an hour they begin to shed copious tears. The nose sympathizes. Y[illegible]r handkerchief suddenly becomes the most important object in life. By the next day the slightest draft of wind sets you to sneezing. It is a revelation. You never before even suspected what it was really to sneeze. If the door is open you sneeze. If a pane of glass is gone you sneeze. If you look into the sunshine you sneeze. If a little dust rises from the carpet, or the odor of flowers is wafted to you, or smell of smoke, you incontinently sneeze. If you sneeze once, you sneeze twenty times. It is a riot of sneezes. First, a single one, like a leader in a flock of sheep bolts over; and then, in spite of all you can do, the whole flock, fifty by count, come dashing over, in twos, in fives, in bunches of twenty.

They do not wait for order. They tread on each other. Four or five sneezes run together—a mere clash of sneezes. Your eyes meanwhile overflow. Your nose rejoices. That has befallen you for which the prophet longed; "Your head has been a fountain of tears." I have heard it said that no one can do well more than one thing at a time. If only you have opthalmic catarrh, you may cry, blow, sneeze and cough each perfectly and altogether. After twenty minutes the paroxysm passes, and you cautiously put up your hands to see if anything is left of your head. It is there! Gradually you get used to it, and come to regard your head as a piece of artillery, invented to throw sneezes.

It is well known that when in health one may stop a sneeze by stroking his nose so as to flatter it a little. There are times when, though a sneeze is a luxury, it is out of place. Much as we like luxuries, we are conscious that they do not fit all seasons or grace all places. One could not willingly eat ice-cream at a funeral, nor be married with a cigar in his mouth.

Lounging on a summer verandah, the bees droning and the flies buzzing, a hearty sneeze is enjoyable. But when a discourse grows touching, and the house is still, and the climax is just reached, a resounding sneeze, especially if you split it up by trying to suppress it, introduces a new train of associations. This is even less agreeable if you are an honorary member, or some distinguished person, put upon the platform, as girls put their pearls or ribbons on the neck, for show.

But just imagine a man in the full fight of a hay-cold going to meeting! and not only, in the words of the immortal Watts, "firing his zeal along the road," but breaking out in sermon-time with a rattle of sneezes—a perfect fusillade! One man sneezing can take an audience out of Whitefield or Demosthenes. Wherefore a sneeze is stronger than an oration.

These are the early symptoms, and continue about a fortnight. Then, without letting go of sneezing, your head becomes stuffed with a real premium cold. You cannot breathe through your nose, and not much through your mouth. You can smell nothing and taste nothing, and see but very little, and that little in water colors. Eating becomes a matter of skill. You cannot eat with your mouth at the same time that you are breathing through it. Two trains meeting on a single track, one or the other must switch off. Thus, you chew and hold your breath ; and then you switch to one side your cud, and breathe a while. Thus you shut off, alternately, bread and breath. This stage lasts about two weeks more. It is not uniform and dull. Now and then a day will, without a cause or warning, plump down on you with all the symptoms of the first period. The stricture will be removed ; eyes and nose, like the hill after winter, will flow down to rills, and sneezes will awaken sylvan echoes, like birds among the trees.

The third stage is known by the tendency of the whole complaint to descend. It seeks to become bronchial. You are seized with spasmodic coughs. If you give way to them, they will leave you feeling as if a blunderbuss, loaded with shot, had been discharged through your lungs. A cough, like a breachy horse, should be ridden with snaffle (not snuffle) and curb, and never suffered to get under way, or to go faster than a walk. But it is not an unrelieved cough, for it alternates with asthma—a complaint in which a man feels, when asleep, as if some one was suffocating him with an unfair use of hemp ; and, when awake, as if the hemp grew inside of him, and he was trying with his breath to pull it up by the roots. Before you had busy days, but now you will have your chief occupation at night.

I do not wish you to think that I have given you all the symptoms. Only the outlines are herein set down. The filling up must be withheld from literatim and left to experience.

One of the striking features of this disorder is the obstinate neglect of the patient to get well.

For remedies are abundant. They are infallible, too. They pour in upon you as thick as rain drops in an equinoctial storm. Each only comes with trophies. It has cured its scores, and can cure everything that sneezes.

Let me draw out a system of treatment from the elements which have been recommended to me. First, a complete routine of water treatment, including hot baths, douches and wet packs, with dripping sheet every morning.

This is to arouse nature in the system and prepare it for thorough work.

Next, as soon as sneezing begins you should procure catarrh snuff, of which there are some forty kinds more or less ; as you cannot be expected to know which one is best adapted to your particular case, you had better try them all. My friend, Mr. Mi[illegible], Fulton street, Brooklyn, will procure them for you with pleasure. He keeps a patent-medicine store near Concord Street, and will be happy to show you in his own person the benefit to be derived from letting medicine alone.

You will now be in a state to use pennyroyal. As it grows everywhere abundantly do not stint yourself. Get an armful. Make a strong tea and drink it ; a strong decoction and wash in it, smell of it, rub your forehead with the leaves, and if you can conveniently, lie down on it and roll as you may have seen a cat do with catnep. Thus far you have been skirmishing. You will now proceed to attack the disease in good earnest. First take two grains of blue pill every night alternately for a week, following it in the morning with a quart bottle of Congress water (every drop must be drunk, the virtue being found as cream in milk, in the strippings.) Mix a pint of sulphur with molasses, and take a teaspoonful morning, noon and night. On alternate days, two grains of quinine may be added three times a day, and ten drops of Fowler's solution (arsenic) continued until a sort of puffness appear about the eyes.

You are now well under way. If you are faithful, signs of faltering will soon appear in your symptoms. You will find them much aggravated. Your spirits will be quite low. But that only shows that the evil spirit is preparing to leave. Redouble your exertions. Get an inhaler and some tincture of cubebs. Fill the former with hot water and put a teaspoonful of the tincture to it, and suck like a new-born babe. Every time your eyes ache pour some tincture of camphor into a bowl of hot water, put a towel over your head, and steam your eyes.

If you can find tuberose blossoms, keep them by you and smell of them often. By this time a little tincture of *lobelia inflata*, taken four times a day, will contribute to the anomalous sensations which you will experience. Put chloride of lime in your sleeping room, and wash your hands and face several times a day in a solution of it.

If now symptoms of asthma appear, take courage! The remedies for asthma are innumerable. Your field is widening! You are now near the end!

It is true, asthma of itself is not a luxury. But it leads you to an acquaintance with the vegetable kingdom, which you might have altogether missed. You will be seen wandering around barn-yards, compost heaps, and old lots filled with builders' debris, hunting for stramonium leaves, to be dried and smoked. You will be seen sitting in a room blue with the smoke of paper saturated with nitre. You will stuff your pockets with troches and such small shot, with which to pepper the enemy. The vegetable poppy, and the plant ipecacuanha, will now become dear to you. Only to think, the ipecac, quinine, and coffee belong to one genus in botany, and all of them good for asthma, except perhaps the two first.

But it is needless for me to extend this discourse of remedies. In such a multitude of them one should be able to pluck health as boys do whortleberries on a New England hill. If the line which I have laid out be faithfully followed, one may expect relief in from eight to ten weeks. If one is unbelieving and will not touch either of them, he will recover in from four to six weeks.

If one subject to the disorder will, about the time of its access, change the air wholly, by a sea voyage, a trip to the mountains—if he lives in the city, or to the city if he lives in the mountains,—he will escape entirely.

I once wrote to Oliver Wendell Holmes as to his knowledge of a remedy. His encouraging reply was;

"Gravel is an effectual cure. It should taken about eight feet deep." HENRY WARD BEECHER.

